# Genetic and Evolutionary Analysis of Purple Leaf Sheath in Rice

**DOI:** 10.1186/s12284-016-0080-y

**Published:** 2016-02-27

**Authors:** Han-shiuan Chin, Yong-pei Wu, Ai-ling Hour, Chwan-yang Hong, Yann-rong Lin

**Affiliations:** Department of Agronomy, National Taiwan University, Taipei, Taiwan; Department of Agronomy, Chiayi Agricultural Experiment Station, Taiwan Agricultural Research Institute, Chiayi, Taiwan; Department of Life Science, Fu-Jen Catholic University, Xinbei, Taiwan; Department of Agricultural Chemistry, National Taiwan University, Taipei, Taiwan

**Keywords:** Genealogy, Genetic Diversity, OsC1, Purple Leaf Sheath

## Abstract

**Background:**

Anthocyanin accumulates in many plant tissues or organs, in rice for example leading to red, purple red and purple phenotypes for protection from damage by biotic and abiotic stresses and for reproduction. Purple leaf, leaf sheath, stigma, pericarp, and apiculus are common in wild rice and landraces and occasionally found in modern cultivars. No gene directly conferring anthocyanin deposited in a purple leaf sheath has yet been isolated by using natural variants. An F_2_ population derived from *ssp. japonica* cv. Tainung 72 (TNG72) with purple leaf sheath (PSH) crossed with *ssp. indica* cv. Taichung Sen 17 (TCS17) with green leaf sheath (GSH) was utilized to isolate a gene conferring leaf sheath color.

**Results:**

By positional cloning, 10-and 3-bp deletions in the R2R3 Myb domain of *OsC1* were uncovered in GSH varieties TCS17 and Nipponbare, respectively. Allelic diversity, rather than gene expression levels of *OsC1*, might be responsible for anthocyanin accumulation. Parsimony-based analysis of genetic diversity in 50 accessions, including cultivars, landraces, and A-genome wild rice, suggests that independent mutation occurred in Asian, African, South American, and Australian species, while *O. meridionalis* had a divergent sequence. *OsC1* was thought of as a domestication related gene, with up to 90 % reduction of genetic diversity in GSH; however, no values from three tests showed significant differences from neutral expectations, implying that *OsC1* had not been subjected to recent selection. Haplotype network analysis revealed that species from different continents formed unique haplotypes with no gene flow. Two major groups of haplotypes corresponding to 10-bp deletion and other sequences were formed in Asian rice, including *O. rufipogon*, *O. nivara* and *O. sativa*. Introgressions of *OsC1* between subspecies through natural and artificial hybridization were not rare. Because artificial and natural selection imposed admixture on rice germplasm in Taiwan, the genealogy of *OsC1* might not be congruent with the current distribution of alleles through lineage diversification.

**Conclusion:**

*OsC1* is responsible for purple leaf sheath, and much new information about *OsC1* is provided e.g., new alleles, non-domestication syndrome, and incongruence of genealogy with geographic distribution.

**Electronic supplementary material:**

The online version of this article (doi:10.1186/s12284-016-0080-y) contains supplementary material, which is available to authorized users.

## Background

Plants accumulate diverse pigments in various tissues and organs related to photosynthesis, defense, and reproduction. Anthocyanins, belonging to the flavonoid class of pigment molecules, are important secondary metabolites in rice (Reddy et al. 1995). Anthocyanin accumulation in different tissues is sometimes involved in many physiological functions, such as modulation of hormone responses, protection from damage by ultra-violet radiation, and defense responses to biotic and abiotic stresses (Reddy et al. 1994; Chalker-Scott 1999; Ithal and Reddy 2004). Purple stigma and apiculus attract insects for pollination and animals for seed dispersal, respectively. Different degrees of purple color are commonly found in the root, leaf sheath, leaf blade, stigma, and apiculus of wild rice species and landraces and occasionally seen in cultivars. Allelic diversity of genes conferring purple pigmentation is maintained in natural germplasm.

Anthocyanin metabolism is regulated by genes and influenced by environmental factors such as pH, ultra-violet radiation and temperature, which have been well studied in maize, *Petunia*, *Arabidopsis* and other model plants (Dooner et al. 1991; Brenda 2001; Koes et al. 2005). In addition to the genes encoding enzymes participating in the anthocyanin synthesis pathway, several regulatory genes including members of the *C1*, *Pl*, *B* and *R* gene families in maize have been identified and their functions elucidated. C1 and Pl proteins containing Myb DNA binding domains regulate downstream genes of the flavonoid synthesis pathway; while B and R proteins containing a basic helix-loop-helix (bHLH) domain are transcriptional activators (Chandler et al. 1989). Based on sequence similarity, *OsC1*, *OsB1* and *OsB2*, which are homologous to maize *C1* and *B*, were isolated from rice (Reddy et al. 1998; Sakamoto et al. 2001). The N-terminal and bHLH domains of OsB1 and OsB2 take advantage of protein-protein interaction with OsC1 that have an R2R3 Myb domain, and these proteins regulate directly the downstream genes of the flavonoid synthesis pathway (Sakamoto et al. 2001; Koes et al. 2005).

Asian rice, *Oryza sativa*, was domesticated from wild species, *O. rufipogon*, an estimated 10,000 years ago. The domestication syndromes are considered to include aspects of grain color, grain size, yield, and other desirable agronomic traits. Domestication-related genes have been classified in terms of crop domestication, improvement and diversification (Kovach et al. 2007; Larson et al. 2014). Shattering genes, *sh4* and *qSH1*, were deliberately modified to influence harvest efficiency as recently as the past 100 years, contributing directly to rice domestication (Zhang et al. 2009). Another key domestication-related gene, *sd*-*1*, resulting in semi-dwarf stature, was a key element of the ‘Green Revolution’ in the 1960s in Asia by virtue of dramatically increasing grain yield (Asano et al. 2007). The purple apiculus trait which is common in wild rice but rare in cultivars is controlled by a regulatory gene, *OsC1*. Allelic diversity of *OsC1* revealed signatures of selection in cultivated Asian rice but not in indigenous improved rice varieties in North India (Saitoh et al. 2004; Choudhury et al. 2014). Nevertheless, some crop improvement traits are regional preferences, such as grain texture and flavor regulated by *waxy* and *BADH2* which harbor allelic diversity in different landraces and cultivars (Olsen and Purugganan 2002; Kovach et al. 2009).

In rice, purple leaf sheath (PSH) as well as purple apiculus and stigma is common in wild species and landraces; however, green leaf sheath (GSH) is prevalent in modern cultivars. One or two major QTLs were suggested to confer leaf sheath color, and two QTLs mapped on chromosomes 1 and 6 could explain more than 50 % of phenotypic variation (Hadagal et al. 1980; Yue et al. 2006). A purple leaf sheath gene, *PSH1* (t), was narrowed down to an interval of 23.5 kb on chromosome 1 encompassing 6 candidate genes after high resolution of linkage mapping. No gene directly conferring PSH has yet been isolated by using natural variants.

In this study, an F_2_ segregating population of ssp. *japonica* cv. Tainung 72 (TNG72) with purple leaf sheath × ssp. *indica* cv. Taichung Sen 17 (TCS17) with green leaf sheath was utilized to isolate a gene conferring leaf sheath color by positional cloning. Four accessions with various levels of anthocyanin accumulation in the leaf sheath were used to investigate the relationship between gene expression and anthocyanin content. Analyses of haplotype and nucleotide diversity based on 50 rice accessions which included improved cultivars, landraces, and wild species were conducted to reveal if the gene has been responsive to artificial selection and the associated geographic distribution of its alleles.

## Result

### Accumulation of Anthocyanins in Tissues of TNG72 and TCS17 at Various Growth Stages

**Fig. 1:**
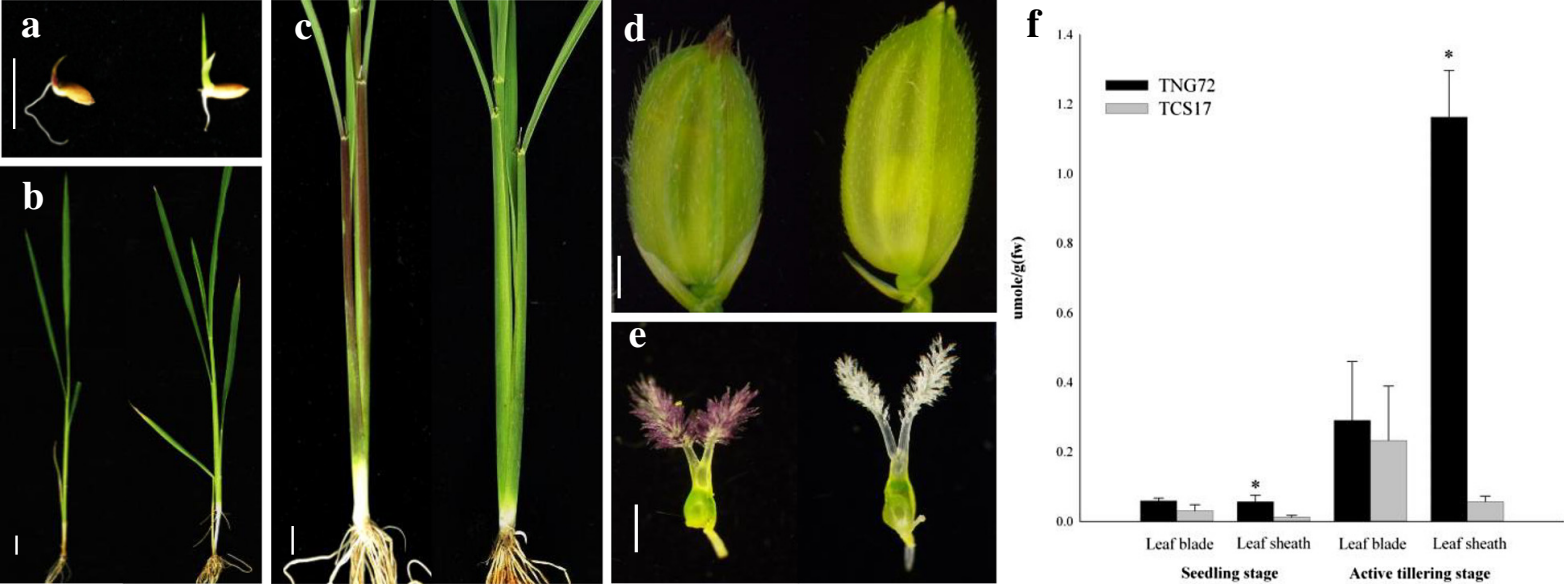
Phenotypes of Tainung 72 (TNG72) and Taichung Sen 17 (TCS17) for different tissues and growth stages. All of the figures are established that left is TNG72 and right is TCS17. Plants at (**a**) coleoptile, (**b**) 4-leaf stage, (**c**) active tillering stage, (**d**) caryopsis and (**e**) stigma at flowering time. Bar size is (**a**) 1 cm; (**b**), (**c**), and (**d**) 5 cm; (**e**) 1 mm. (**f**) Estimated anthocyanin contents in leaf sheath and blade at seedling and active tillering stages. * *p* < 0.05 using paired *t*-test

TNG72 possessed purple coleoptile, leaf sheath, apiculus, and stigma, and the degrees of purple color varied at different growth stages (Fig. 1a-e). The leaf sheath of TNG72 was green at the 4-leaf stage but gradually turned purple until the active tillering stage, remaining purple until maturity. TNG72 accumulated various amounts of anthocyanin with different localization in rice tissues. However, TCS17 remained green for the entire life cycle, representing the most common phenotype of cultivated rice (Fig. 1a-e).

Both TNG72 and TCS17 possess green leaf blades for their entire life. The anthocyanin contents of TNG72 and TCS17 leaf blades did not differ significantly although TNG72 was somewhat higher at both seedling and tillering stages (Fig. 1f) and may have already had anthocyanins at a level too low to be discerned visually. On the other hand, TNG72 had significantly higher (*p* < 0.05) anthocyanin content in leaf sheaths than TCS17. Leaf sheath anthocyanin contents were estimated as 0.06 μmol/g and 0.01 μmol/g at the 4-leaf seedling stage; and 1.16 μmol/g and 0.05 μmol/g at active tillering for TNG72 and TCS17, respectively (Fig. 1f).

### Isolation of the Gene Conferring the Leaf Sheath Color

**Fig. 2:**
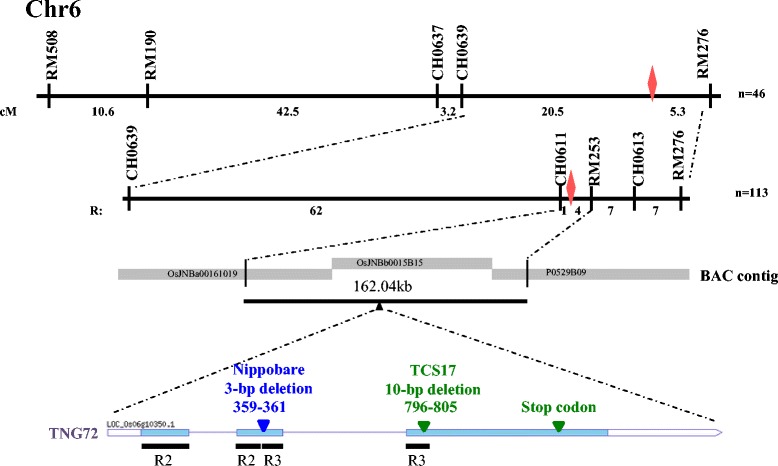
Isolation and sequence analysis of the gene conferring responsible for purple leaf sheath. Coarse mapping of the purple leaf sheath gene between CH0639 and RM276 on chromosome 6. Fine mapping narrows down this region to 160.04 kb between CH0611 and RM253. The *LOC*_*06g10350* locus was annotated as MYB family transcription factor by RGAP and corresponded to *Os06g0205100* annotated as transcription factor MYB6 and *O. sativa* C1, and rice homolog of maize C1 by RAP. Sequence analysis of 3 cultivars, Tainung 72 (TNG72), Nipponbare and Taichung Sen 17 (TCS17). TNG72 has complete sequences translated into 272 amino acids. Nipponbare has 3-bp deletion in the second exon of *OsC1*, and is translated into 271 amino acids. TNG17 has 10-bp deletion in the third exon of *OsC1*, and is translated into 207 amino acids. The black lines are conserved domains of *OsC1*, R2R3 Myb domain. The triangles indicate sites of variation

The F_2_ population of TNG72 ×TCS17 was used to identify the gene conferring leaf sheath color by positional cloning. A total of 632 F_2_ progenies included 473 and 159 individuals exhibited PSH and GSH, respectively. The segregation ratio of 2.9:1 (PSH: GSH) followed single-gene Mendelian inheritance, indicating that GSH was recessive. A randomly selected 46 F_2_ plants with green leaf sheath were genotyped with 117 polymorphic markers distributed over the 12 rice chromosomes, permitting the gene to be coarsely mapped between CH0639 and RM276 on the short arm of chromosome 6 (Fig. 2). An additional 113 F_2_ plants with GSH were genotyped with 3 more markers for fine mapping. The target interval was 162.04 kb, between CH0611 and RM253 encompassed by 3 BACs, OsJNBa00161019, OsJNBb0015B15, and P0529B09. By retrieving the Rice Genome Annotation Project database (RGAP, http://rice.plantbiology.msu.edu), it was found that one of 25 candidate genes, *Os06g10350*, annotated as a MYB family transcription factor, was the most likely gene. *Os06g10350* corresponded to *Os06g0205100*, annotated as transcription factor MYB6, *O. sativa* C1, a rice homolog of maize C1 in the Rice Annotation Project database (RAP, http://rapdb.dna.affrc.go.jp/).

TNG72 with PSH possessed a full-length *OsC1* DNA sequence encoding 272 amino acids. TCS17 with GSH had a 10-bp deletion in the R3 Myb domain, which was considered a core domain in exon 3 of *OsC1*. The 10-bp deletion resulted in a frame shift, translating only 207 amino acids and leading to a premature stop codon. In the reference genome sequence of Nipponbare on RGAP, a 3-bp deletion of part of the R3 Myb domain in exon 2 was noted, for which Nipponbare has GSH. The results revealed that an aberrant R3 Myb domain of anthocyanin regulatory C1 protein caused a deficit in anthocyanin synthesis in TCS17 and Nipponbare.

### Natural Variation in Leaf Sheath Colors

Purple leaf sheath is a common phenotype in wild rice. In this study, among the 7 wild rices utilized in the *Oryza* Map Alignment Project (OMAP), all except *O. nivara* (103813) exhibit PHS. One accession of *O. rufipogon* collected from Taiwan also has purple leaf sheath. Nevertheless, the majority of cultivated rice has GSH. Among the 43 accessions of *O. sativa* selected to study genetic diversity of *OsC1*, 17 *indica* and 12 *japonica* accessions possess green leaf sheath, while 5 *indica* and 9 *japonica* accessions possess purple leaf sheath (Table 1). Because of the intention to study allelic variation of *OsC1*, more *japonica* landraces exhibiting various degrees of purple color on the leaf sheath were included.

**Table 1:** A total of 50 accessions for nucleotide diversity analysis

Taxon	Accession	Origin	Color of leaf sheath
*O. sativa* ssp. *indica*			
cultivars	Tainung Sen 20	Taiwan	Purple
	Jianungyu 892229	Taiwan	Green
	Jianungyu 892234	Taiwan	Green
	Taichung Sen 17	Taiwan	Green
	Taichung Native 1	Taiwan	Green
	IR 13525-118-3-2-2-2	The Philippines	Purple
	IR64	The Philippines	Green
	Hua Keng Sen 7	China	Green
	Hu Han 15	China	Green
landrace	Cheng Ching Yu	Taiwan	Green
	Cheng Wu Chan	Taiwan	Green
	Chu Tzu	Taiwan	Green
	Dee Geo Woo Gen	Taiwan	Green
	Hua Lou	Taiwan	Green
	Midon	Taiwan	Green
	Jinya-149	Taiwan	Purple
	G 124	India	Purple
	Shui Pai Tiao	China	Purple
	Fu P’I Sen	China	Green
	Hsia Men Chung	China	Green
	Liu Shih Jih Tsao	China	Green
	Yin Yu Tzu	China	Green
*O. sativa* ssp. *japonica*			
cultivars	Tainung 72	Taiwan	Purple
	Taichung 65	Taiwan	Green
	Tainung 67	Taiwan	Green
	Shinriki	Japan	Green
	Asamurasaki	Japan	Purple
	Kameji	Japan	Green
	Nipponbare	Japan	Green
	Nohrin 1	Japan	Green
landrace	Chuan4	Taiwan	Purple
	Kun Shan Wu Siang Keng	Taiwan	Purple
	Tongsisai	Taiwan	Purple
	Warisanmochi 2	Taiwan	Purple
	Baridon	Taiwan	Green
	Chien Tzu Chu	Taiwan	Green
	Ch’ih K’o	Taiwan	Green
	Nobohai	Taiwan	Green
	Munagurusu	Taiwan	Green
	Ssall-Bye	Taiwan	Purple
	4233	China	Purple
	Shang Chi Tsao Tao	China	Purple
	Yen No	China	Green
*O. barthii*	10412	Cameroon	Purple
*O. glaberrima*	96717	Senegal	Purple
*O. glumaepatula*	105668	Brazil	Purple
*O. meridionalis*	105300	Australia	Purple
*O. nivara*	103813	China	Green
*O. nivara*	104683	India	Purple
*O. rufipogon*	Taiwan type 1	Taiwan	Purple

**Fig. 3:**
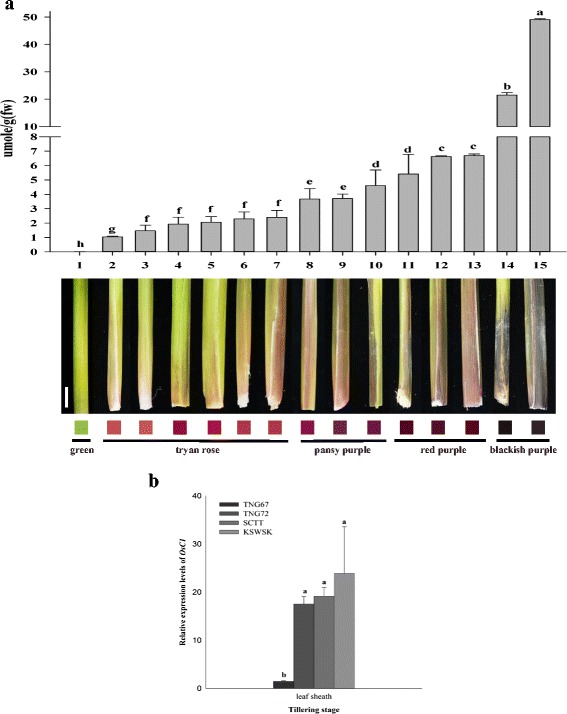
Anthocyanin accumulation and gene expression of *OsC1* in the leaf sheath. (**a**) Phenotypes and anthocyanin contents of 1 green leaf sheath (GSH) accession and 14 purple leaf sheath (PSH) accessions with various degrees of purple. The 15 accessions are Tainung 67 (TNG67) (1), Chuan4 (2), G124 (3), Warisanmochi2 (4), Shui Pai Tiao (5), Tonsisai (6), IR13525-118-3-2-2-2 (7), 4233 8 (8), Jinya-149 (9), Tainung 72 (TNG72) (10), Ssall-Bye (11), Tainung Sen 20 (12), Shang Chi Tsao Tao (SCTT) (13), Asamurasaki (14), and Kun Shan Wu Siang Keng (KSWSK) (15). Leaf sheath phenotypes are characterized according to the RHS (Royal Horticultural Society, 5th edition) Color Charts. (**b**) Gene expression of *OsC1* in the leaf sheath of 4 cultivars at tillering. Cultivars TNG67, TNG72, SCTT and KSWSK, exhibiting diverse degrees of purple color, were selected to evaluate gene expression by real-time PCR. Letters indicate differences at significance level *p* < 0.05 by using LSD analysis

Various degrees of purple color were obvious among different rice accessions. Fourteen accessions, including 2 cultivars and 3 landraces of *indica* rice, and 1 cultivar and 8 landraces of *japonica* rice, ranged from light to dark purple. According to the RHS (Royal Horticultural Society) Color Charts 5th Edition, the leaf sheath of 6, 3, 3, and 2 accessions showed tyran rose, pansy purple, red purple, and blackish purple, respectively (Fig. 3a). The estimated anthocyanin content of the GSH cultivar, Tainung 67 (TNG67), was 0.03 μmol/g. The anthocyanin contents of 14 PSH ranged from 1.04-42.77 μmol/g, for which Chuan4 with tyran rose had the least while Kun Shan Wu Siang Keng (KSWSK) with blackish purple had the most. The 14 accessions could be divided into 7 groups with significant differences (LSD, *p* ≤0.05), in which color degrees were in proportion to anthocyanin contents (Fig. 3a).

One *japonica* GSH accession, TNG67, and 3 *japonica* PSH accessions, TNG72, Shang Chi Tsao Tao (SCTT) and KSWSK, exhibiting different degrees of purple color in the leaf sheath were used to evaluate gene expression of *OsC1*. At the active tillering stage, the estimated anthocyanin contents of these four accessions were significantly different (Fig. 3a). The relative gene expression of 3 accessions with purple leaf sheath was 18-fold higher than TNG67, a highly significant difference (Fig. 3b). Nevertheless, there were no significant differences among the 3 PSH accessions by using LSD analysis.

### Nucleotide Diversity and Haplotype Network of *OsC1*

**Fig. 4:**
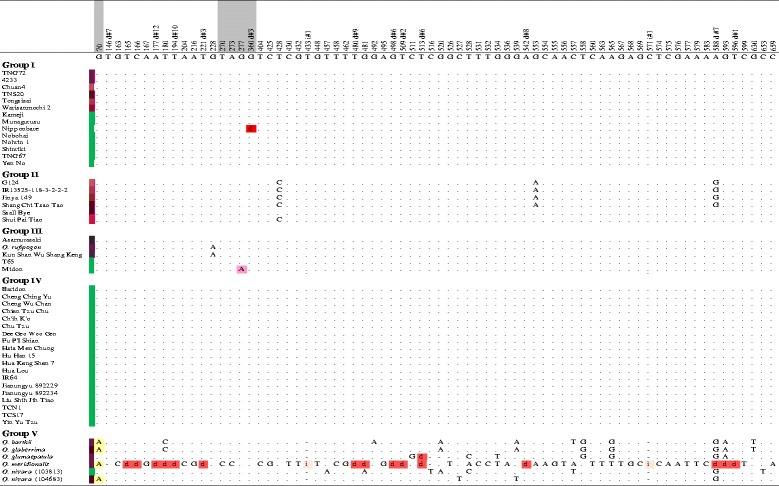
Sequence variation of *OsC1* in 50 accessions. According to DNA sequence variation, 5 groups were classified. The accessions of Group I exhibit red purple, purple, tryan rose and green leaf sheath and have complete *OsC1* sequences closely resembling that of Tainung 72. The accessions of Group II have red purple and tryan rose leaf sheath and share the sequence of Shang Chi Tsao Tao, which has one nonsynonymous mutation in position 1192. The accessions of Group III have blackish purple and green leaf sheath and share the sequence of Kun Shan Wu Siang Keng, which has one nonsynonymous mutation in position 918. The accessions of Group IV have green leaf sheath and share the sequence of Taichung Sen 17, which has a 10-bp deletion starting at position 796. The accessions of Group V include 6 wild A-genome species. Non-synonymous amino acid mutations were indicated to have similar function (yellow) or altered polarity (blue), charge (pink) and aromatic nature (green). d: deletion. i: insertion

Fifty accessions collected from 9 countries, mainly from Taiwan, were subjected to analysis of *OsC1* nucleotide variation and its haplotype network (Table 1). These 50 accessions could be classified into 5 groups, with Groups I-IV sharing high similarity of DNA sequence while Group V including 6 A-genome wild rices exhibited more allelic diversity (Fig. 4). For Group I, all 14 accessions shared high sequence similarity with TNG72. The majority of Group I belonged to ssp. *japonica* except 1 *indica* cultivar, Tainung Sen 20. The leaf sheaths of three accessions were tyran rose or pansy purple. Nevertheless, 8 accessions had GSH although *OsC1* function was predicted to be normal. Group II, consisting of 4 *indica* and 2 *japonica* accessions, shared the same sequence as SCTT. There were 5 SNPs that differentiated the group from the *OsC1* sequence of TNG72, and one SNP located at position 1,192 bp in exon 3 conferred nonsynonymous substitution of valine to alanine. All 6 accessions had PSH with colors of tyran rose, pansy purple, and red purple. Group III, including 5 accessions, had one SNP in common at position 918 bp on exon 3, resulting in nonsynonymous substitution from proline to glutamine. All 5 accessions shared high sequence similarity to KSWSK. Three accessions, including 2 *japonica* landraces and *O. rufipogon* Taiwan Type 1, had blackish purple leaf sheath; while the other 2 had green leaf sheath. Group IV, consisting of 16 *indica* and 3 *japonica* accessions, had the same 10-bp deletion at position 796–805 bp as TCS17, causing frameshift mutation. All 19 accessions had green leaf sheath. Group V consisted of all A-genome wild rices except *O. rufipogon* Taiwan Type 1. The nucleotide sequences of the 6 wild rices were highly diverse, especially that of *O. meridionalis*. Five of the 6 wild rices had PSH with various degrees of purple color but *O. nivara* (103813) had GSH (Fig. 4).

**Fig. 5:**
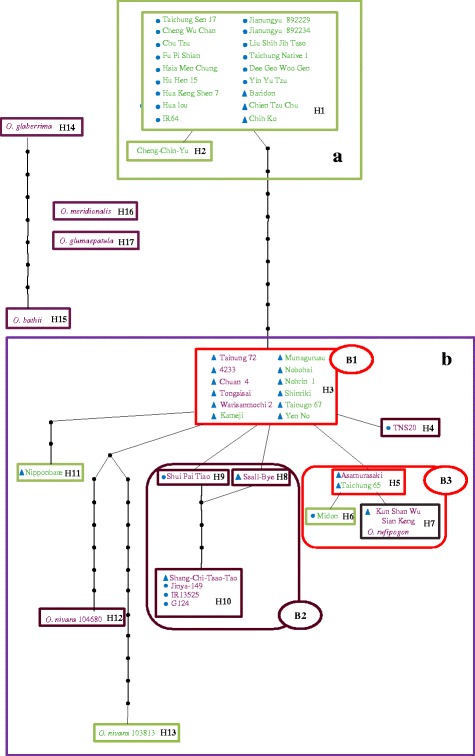
Haplotype network analysis for *OsC1*. A total of 17 haplotypes, H1-H17, were discerned, and each is represented by a rectangle. In Asian rice, two major groups, Group **a** and Group **b**, are classified. Group **b** can be subdivided into three clusters, B1, B2, and B3, indicated by ovals. Blue dots and triangles indicate *indica* and *japonica* accessions, respectively. Text color resembles the color of leaf sheath (green, purple). Box line color resembles the color of leaf sheath of majority of accessions, green, pansy purple, red purple, and blackish purple, respective, besides red resembles admixture of different colors. Black nodes represent inferred branch on which each single nucleotide polymorphism occurred

Haplotype network analysis of *OsC1* gene sequences of these 50 accessions revealed 17 haplotypes with four major distinct groups comprised of Asian, African, Australian, and South American rice, in agreement with genealogical lineages. The 43 accessions of Asian cultivated rice and their wild progenitors, 2 accessions of *O. nivara* and 1 accession of *O. rufipogon*, formed 13 interconnected haplotypes. The African cultivated species *O. glaberrima* and its wild progenitor, *O. barthii*, had different haplotypes, H14 and H15; however, these two haplotypes were connected. *O. meridonalis* (from Australia) and *O. glumaepatula* (South America) each formed independent unique haplotypes, H16 and H17, respectively (Fig. 5).

*OsC1* in the 7 A-genome wild rices, as expected, exhibited rich nucleotide diversity, especially in *O. meridonalis*, as revealed by haplotype number (H), haplotype diversity (H_d_), polymorphic sites (S), pairwise nucleotide difference (π_T_, π_sil_), and Watterson’s estimators of θ_T_ and θ_sil_ (Table 2). Nucleotide diversity of *OsC1* in cultivated *O. sativa* was apparently reduced. In general, more genetic variation was detected in *indica* than *japonica* rice. Greater sequence polymorphism was noted in PSH than GSH accessions in both *indica* and *japonica* rice despite characterizing more GSH accessions. Up to 90 % reduction of genetic diversity in GSH was revealed by the average number of pairwise nucleotide differences per site based on the total number of polymorphic sites (π_T_); however, less reduction in silent sites was indicated by π_sil_ and θ_sil_ (Table 2).

**Table 2:** Nucleotide divergence of *OsC1*

Taxon	N	H	H_d_	S	π_T_	θ_T_	π_sil_	θ_sil_
O. sativa	43	10	0.481	11	0.00104	0.00196	0.00146	0.00206
spp. *indica*	23	7	0.522	10	0.00129	0.00208	0.00163	0.00201
purple leaf sheath	5	4	0.900	7	0.00275	0.00257	0.00325	0.00284
green leaf sheath	18	3	0.216	3	0.00026	0.00067	0.00016	0.00043
spp. *japonica*	20	5	0.442	7	0.00074	0.00152	0.00127	0.00251
purple leaf sheath	9	5	0.722	7	0.00144	0.00196	0.00246	0.00326
green leaf sheath	11	2	0.182	1	0.00014	0.00026	0.00027	0.00051
Total numbers of purple leaf sheath	14	8	0.857	9	0.00229	0.00216	0.00336	0.00279
Total numbers of green leaf sheath	29	4	0.200	3	0.00021	0.00059	0.00020	0.00038
A-genome wild rice	7	7	1.000	94	0.02584	0.03478	0.03568	N.A.
A-genome wild rice without O. meridonalis	6	6	1.000	37	0.01283	0.01325	0.01544	0.01612

*N* total number of sequences, *H* total number of haplotypes at individual loci in each taxon, *H*_*d*_ haplotype diversity, *S* total number of polymorphic sites, π_*T*_ average number of pairwise nucleotide difference per site calculated based on the total number of polymorphic sites, θ_*T*_ Watterson’s estimator of θ per base pair calculated based on the total number of polymorphic sites, π_*sil*_ average number of pairwise nucleotide difference per site calculated based on silent sites, θ_*sil*_ Watterson’s estimator of θ per base pair calculated based on silent sites, *N.A*. Not applicable. When the proportion of differences is higher than 0.75, the Jukes and Cantor correction cannot be computed

Tajima’s D and Fu and Li’s D & F were applied to test whether *OsC1* deviated from the neutral expectation of heterozygosity, but no values were statistically significant, indicating no evidence of strong selection (Table 3). The overall values were negative in *O. sativa* and the other A-genome species. Positive values, implying balancing selection, were noted in *indica* PSH accessions but not in *japonica* PSH accessions. Negative selection or purifying selection acting on *OsC1* could not be ruled out in *indica* GSH accessions (0.01 ≤ *p* ≤ 0.05).

**Table 3:** Neutrality tests of *OsC1*

Taxon	N	Tajima’s D	Fu and Li’s D	Fu and Li’s F
O. sativa	43	−1.41027	−1.32676	−1.59411
spp. *indica*	23	−1.27639	−1.07742	−1.32111
purple leaf sheath	5	0.49788	0.49788	0.51896
green leaf sheath	18	−1.71304^*^	−2.30153^*^	−2.45793^*^
spp. *japonica*	20	−1.69169^*^	−1.82926	−2.06967
purple leaf sheath	9	−1.18930	−1.02031	−1.18277
green leaf sheath	11	−1.12850	−1.28946	−1.39919
Total number of purple leaf sheath	14	0.23043	0.02189	0.08865
Total number of green leaf sheath	29	−1.53343	−1.45773	−1.71236
A-genome wild rice	7	−1.33215	−1.32405	−1.46956
A-genome wild rice without O. meridonalis	6	−0.22043	−0.17585	−0.20295

^*^The values of Tajima’s D test and Fu and Li’s D&F test are at significance levels of 0.10 < *P* < 0.05

Asian rice could be divided two major subgroups, with Group A including two haplotypes (H1, H2) and Group B including 11 haplotypes (H3-H13) (Fig. 5). All accessions of Group A exhibited GSH, and haplotype H1 containing 15 *indica* accessions and 3 *japonica* landraces all had the same 10-bp deletion. One *indica* landrace, Cheng Ching Yu, has an additional single nucleotide substitution, resulting in another haplotype, H2 (Figs. 4 and 5). Group B contained 27 accessions, 11 GSH and 16 PSH with various degrees of purple. According to the haplotype network, 3 major clusters, B1-B3, could be resolved within Group B. The 12 accessions of Cluster B1 shared the same sequence with PSH TNG72 but 7 were phenotypically GSH. All 6 accessions of Cluster B2 (4 *indica* and 2 *japonica* landraces), expressed pansy purple and red leaf sheath, similar to SCTT. Cluster B3 had 2 GSH and 3 PSH accessions sharing high sequence similarity with KSWSK. Interestingly, *O. rufipogon* (Taiwan Type 1) and KSWSK were embodied in one haplotype, H7. The *indica* cultivar TNS20 and the *japonica* cultivar Nipponbare had their own additional mutations and were separated into two distinct haplotypes, H4 and H11, respectively. The two accessions of *O. nivara* formed different haplotypes, H12 and H13, and were split from Cluster B1 because of diverse nucleotide sequences in both upstream and genic regions (Figs. 4 and 5).

## Discussion

### Allelic Diversity of *OsC1* Responsible for Variation in Leaf Sheath Colors

Plants accumulate anthocyanin in various tissues as an aid to survival and reproduction. In rice, anthocyanin is deposited in the root, leaf sheath, internode, leaf blade, lemma, palea, apiculus, stigma, and pericarp. Tissue-specific anthocyanin accumulation is common in numerous genotypes. Purple pigmented traits in different tissues do not always co-segregate (Sakamoto et al. 2001). However, purple leaf sheath, apiculus, and stigma cosegregated in the F_2_ population of PSH TNG72 × GSH TCS17 (Fig. 1) and other populations (Fan et al. 2008; Gao et al. 2011). Anthocyanin accumulation in the apiculus had previously been related to *OsC1* (Takahashi 1957; Saitoh et al. 2004) but in the leaf sheath was thought to be inherited by polygenes or a single gene (Fig. 2; Fan et al. 2008; Wang et al. 2009; Gao et al. 2011).

*OsC1*, conferring leaf sheath color, was isolated from the F_2_ population of TNG72 × TCS17 by positional cloning (Fig. 2), differing from *PSH1* (t) but the same as a locus identified from the somaclonal line Z418 (Wang et al. 2009; Gao et al. 2011). The PSH allele was dominant to GSH in crosses between natural germplasm or mutant lines. PSH TNG72 had a full length *OsC1* allele encoding 272 amino acids while GSH TCS17 had a 10-bp deletion in the R3 Myb domain in exon 3, resulting in truncated translations of 207 amino acids. GSH cultivar Nipponbare had another mutated allele, a 3-bp deletion in the R3 Myb domain in exon 2 (Fig. 2). Among the 50 accessions analyzed herein, the 10-bp deletion was found in 17 *indica* landraces and improved cultivars from Taiwan, China, and the Philippines, and 2 *japonica* landraces from Taiwan (Fig. 4). The 10-bp deletion was conserved in 17 indigenous varieties in Northeast India and only observed in *indica* varieties from Taiwan, China, India, and Indonesia (Saitoh et al. 2004; Choudhury et al. 2014).

Ten GSH accessions without the 10-bp deletion of *OsC1* had other mutated alleles or genes, leading to absence of anthocyanin pigmentation. Deletions of 3-bp in exon 2 and 2-bp in exon 3, both in the R3 Myb domain, were found in *japonica* rice from Japan and China, respectively (Figs. 2 and 4; Saitoh et al. 2004). Two GSH accessions, Midon and T65, had an amino acid substitution at position 918. However, three other accessions with the same substitution, Asamurasaki, KSWSK, and *O. rufipogon*, had blackish purple leaf sheath. Thus, mutations in genes other than *OsC1* were suggested to also eliminate anthocyanin pigmentation, a hypothesis that was supported by seven *japonica* accessions having the same *OsC1* coding sequence as PSH TNG72 (Fig. 4). *OsC1*, containing an R2R3 Myb domain, is thought to function as a transcription factor, regulating other genes involved in anthocyanin synthesis and enhancing another transcription factor, bHLH, regulating anthocyanin structural gene DFR (Dooner et al. 1991; Ithal and Reddy 2004). Failure of anthocyanin synthesis could result from malfunction of *OsC1* or any downstream proteins: for example, *PSH1* (t) conferring purple leaf sheath was mapped on chromosome 1 (Wang et al. 2009). One of two activator genes, *OsB1* and *OsB2* encoding basic helix-loop-helix (bHLH) transcription factors, incorporated with maize *C1* could induce anthocyanin synthesis in the aleurone layer; however, the lack of function of these two genes in T65 resulted in green leaf blade and leaf sheath (Sakamoto et al. 2001). One *japonica* GSH accession, TNG67 which is a descendant of T65, had significantly reduced expression of *OsC1* despite having a full length amino acid encoding sequence (Fig. 3), suggesting feedback regulation of gene expression.

Fourteen PSH accessions exhibited variation in intensity of purple coloring of the leaf sheath, classified as tyran rose, pansy purple, red purple, and blackish purple. The anthocyanin contents of these 14 PSH accessions differed significantly (Fig. 3). However, *OsC1* gene expression among three accessions with diverse anthocyanin contents, TNG72, SCTT, and KSWSK, showed only slight and non-significant differences. These three accessions represented three different haplotypes, H3, H7, and H10, respectively (Fig. 5). Allelic variation of *OsC1* accounted for anthocyanin accumulation and pigmentation in the leaf sheath, for which nonsynonymous mutations at the C-terminal domain were found herein. The C terminus of R2R3 Myb transcription factors is an activation and repression domain (Dubos et al. 2010). Allelic variation of *OsC1* and *Purple leaf* (*Pl*) coding regions also caused diverse intensities of purple apiculus pigmentation (Sakamoto et al. 2001; Saitoh et al. 2004). Allelic variation resulting in diversified phenotypes is very common, as exemplified by the great impact of different alleles of *Wx* and starch synthesis related genes on rice grain appearance, cooking and eating quality (Tian et al. 2009; Zhang et al. 2012; Wu et al. 2015). However, other genes participating in anthocyanin synthesis and environmental effects cannot be neglected (Sakamoto et al. 2001). Anthocyanin accumulation is frequently responsive to abiotic stresses, such as UVB radiation, temperature, and soil acidity (Reddy et al. 2004). A *cis*-element related to light regulation (−10PEHVPSBD, Thum et al. 2001) was found in the promoter region of *OsC1* in TNG72 by using the PLACE database (https://sogo.dna.affrc.go.jp/, Higo et al 1999). We noted that the leaf sheath accumulated more anthocyanin when rice plants were grown in soil under natural light than in hypotonic solution in the growth chamber.

### The Selection and Genealogy of *OsC1*

Traits considered part of domestication syndromes are favored by artificial selection during domestication, and consequently distinguish cultivated plants from their wild progenitors. During domestication, genetic diversity of whole genomes can be dramatically reduced because of the ‘genetic bottleneck’ effect of selecting a few individuals as a founder population. The genetic diversities of genes contributing to domestication syndromes and improvement traits are often accompanied by artificial selection for production and culture, and the geographic distribution of alleles might be altered by human migration (Kovach et al., 2007; Kovach and McCouch 2008; Olsen and Wendel 2013). In rice, *OsC1* conferring purple leaf sheath, apiculus, and stigma has been suggested as a domestication gene (Choudhury et al. 2014). Wild types are generally purple while cultivars tend to be green, although exceptions exist as noted above. The nucleotide diversity of *OsC1* was higher in *O. rufipogon* than in *O. sativa*, and *OsC1* of the haplotype of Asian cultivated accessions showed evidence of selection (Saitoh et al. 2004). In addition to perennial *O. rufipogon* and annual *O. rufipogon* (also called *O. nivara*), *OsC1* sequences of 4 other wild species, *O. glaberrima*, *O. barthii*, *O. glumaepatula*, and *O. meridionalis* which had not been previously reported were subjected to allelic variation analysis. Sequence variation existed both in noncoding and coding regions. While these species had PSH, a few nonsynonymous amino acid substitutions were detected (Fig. 4). *O. meridionalis* (from Australia) had extremely divergent *OsC1* DNA sequence, forming a distinct haplotype by itself, and all parameters regarding nucleotide divergence of *OsC1* decreased when it was not included in analysis (Table 2). African cultivated species, *O. glaberrima*, its progenitor *O. barthii*, and South American species, *O. glumaepatula*, also had their own specific sequences and formed 2 different haplotypes (Figs. 4 and 5). On the other hand, *OsC1* in Asian rice shared sequence similarity and formed 13 interconnected haplotypes. Allelic variation of *OsC1* in Asian, African, South American, and Australian rice reflected independent mutation without gene flow because of their geographic distribution. Two accessions of *O. nivara* and one accession of *O. rufipogon* together with 24 accessions of *O. sativa* were in Group B. The genealogy of *OsC1* in the collected Asian rice accessions herein and other studies showed no distinct correlation with geographic distribution. The 10-bp deletion conferring GSH was prevalent in *indica* landraces and improved cultivars and rare in *japonica* accessions from many countries (Saitoh et al. 2004; Choudhury et al. 2014). The accessions in haplotypes H3 and H10 were collected from several countries (Fig. 5). Gene flow due to human activities might be an important factor in the geographic distribution of *OsC1* alleles.

Purple leaf sheath, stigma, and apiculus are widespread in wild forms and often found in landraces, while green leaf sheath as well as colorless stigma and apiculus are common in modern cultivars. *OsC1* nucleotide divergence was up to 90 % lower in GSH than PSH although twice as many GSH accessions were studied (Table 2). However, no values from three neutral tests, Tajima’s D, Fu and Li’s D & F, were significantly differently from neutral expectations, indicating that *OsC1* had not been subjected to selection–a conclusion that is supported by findings regarding *OsC1* in indigenous rice varieties in Northeast India (Choudhury et al. 2014). Genetic diversity was higher in *indica* than *japonica* accessions both in 14 PSH and 20 GSH accessions, revealed by parameters reflecting nucleotide segregation at total polymorphic sites and silent sites, π_T_, θ_T_, π_sil_, and θ_sil_ (Table 2, Fig. 4). This phenomenon is congruent with evidence that genetic diversity is larger in subspecies *indica* than *japonica* (Garris et al. 2005).

Selection might not be a driving force for reducing genetic diversity in GSH. There is no significant evidence that *OsC1* deviated from neutral expectations in *indica* PSH or *japonica* PSH and GSH accessions. Nevertheless, *indica* GSH accessions might be experiencing relaxed purifying selection, indicated by neutrality tests at significance levels of 0.10 < *P* < 0.05 (Table 3). Unlike other domestication syndromes directly related to productivity and other desirable traits that were selected for particular purposes over several thousand years, GSH might have been selected unintentionally.

The Asian cultivated species, *O. sativa*, evolved from Asian wild rice progenitors *O. nivara* (annual) and *O. rufipogon* (perennial). *O. sativa* was domesticated from divergent wild populations about 10,000 years ago and diversified into two major subspecies, *indica* and *japonica*, subsequently being subjected to a long period of natural and artificial diversifying selection (Gross and Zhao 2014). *Indica* and *japonica* subspecies are distinguishable in morphology and physiology, already recognized as Hsien (long grain) and Keng (short grain) in the Han dynasty, China, over 2,000 years ago (Oka 1988; Callaway 2014). Numerous genes related to differentiation between these two subspecies experienced mutation and diversifying selection, e.g., *Phr1* responsible for phenol reaction; and *GS3*, *qSW5* and *GS5* responsible for grain shape (Yu et al. 2008; Lu et al. 2013). The 10-bp deletion conferring GSH was prevalent in *indica* landraces and improved cultivars and rare in *japonica*, but not in its progenitor species. The *OsC1* allele with 10-bp deletion was suggested to have originated and been an early target of domestication in subspecies *indica* (Figs. 4 and 5; Saitoh et al. 2004; Choudhury et al. 2014). In addition, 3-bp and 2-bp deletions in exon 2 and exon 3 of the R3 Myb domain were found in *japonica* rice from Japan and China, respectively. These two alleles were independent from the gene lineage of *indica*, which suggested mutation after subspecies divergence (Figs. 2 and 4; Saitoh et al. 2004).

Although reproductive barriers such as hybrid sterility and hybrid breakdown impede gene flow between cultivated rice and its wild progenitors, numerous interspecific crosses and successful introgressive hybridizations have been performed to unravel useful alleles and genes of wild species. Gene flow confounded with selection has been revealed at the genome level (Zhao et al. 2010; He et al. 2011; Yang et al. 2011) and in domestication-related genes including *Wx*, *GS3*, *SD1*, and *qSH1* (Yamanaka et al. 2004; Konishi et al. 2006; Takano-Kai et al. 2009; Asano et al. 2011).

The genealogy of *OsC1* suggests some gene flow events. *O. rufipogon* (Taiwan type 1) had an *OsC1* sequence identical to that of Taiwan landrace KSWSK. One SNP aligned to *japonica* Asamursaki and Taichung 65 and *indica* Midon, accessions that were clustered in Group III and haplotype B3 (Fig. 4 and 5). Two *O. nivara* accessions also shared similar sequences to most *japonica* accessions and were classified as haplotype B1. Thus, *OsC1* in three Asian wild accessions closely resembled that of *japonica* but not *indica*, a finding which might support the hypothesis that *japonica* and *indica* were domesticated independently from *O. rufipogon* (Yang et al. 2011; Wei et al. 2014). Gene flow between subspecies was not rare, as revealed by genealogy of *OsC1* in 23 *indica* and 20 *japonica* accessions. In haplotype H1, three *japonica* landraces from Taiwan also possessed the 10-bp deletion specific to *indica*; in haplotype H10, one *japonica* landrace (Shang Chi Tsao Tao) and three *indica* accessions (Taiwan landrace Jinya-149, India landrace G124 and improved line IR1535) had the same allele (Figs. 4 and 5).

The genealogy of *OsC1* might not be in agreement with rice phylogeography because of human behavior. In Taiwan, *O. rufipogon* and *O. nivara* were once found in several swamp sites (Chang 1976) but, unfortunately, all habitats were destroyed several decades ago. Archaeological evidence shows that tropical *japonica* or *indica* had been cultivated over 5,000 years by ancient indigenous peoples in Taiwan (Hsieh et al. 2011). In the early 17th century, numerous Chinese migrated and carried many landraces (mostly *indica*) from coastal regions of Fujian and Guangdong Provinces of China to Taiwan. By the early 20th century, 1,197 *indica* accessions were identified officially in Taiwan, and 1,256 *japonica* accessions were introduced from Japan (Iso 1964). More than 1,000 of these accessions were deposited in The T.T. Chang Genetic Resources Center at the International Rice Research Institute (IRRI), the Philippines. Taiwanese rice germplasm was thus an admixture of indigenous wild species, landraces, and introduced germplasm from China and Japan; and was subsequently spread over Southeast Asia via the germplasm deposited in IRRI. As a result, introgression of *OsC1* may have occurred by hybridization between subspecies and both artificial and natural selection, clouding the true genealogy of *OsC1*.

## Conclusion

We used positional cloning to isolate a PSH gene, revealing independent 10-and 3-bp deletions in the R2R3 Myb domain of *OsC1* that occurred in different lineages. Allelic diversity resulted in different *OsC1* protein function rather than gene expression levels, accounting for various degrees of anthocyanin accumulation and associated intensities of purple color. Allelic variation of *OsC1* among wild species, landraces, and cultivars revealed greatly reduced genetic variation in GSH phenotypes, but there was little evidence that *OsC1* had experienced recent selection, suggesting that it may have been enriched in cultivated forms by chance. Introgressions of *OsC1* between subspecies *indica* and *japonica* were frequent through natural and artificial hybridization. Because of admixture of rice germplasm by artificial and natural selection in Taiwan, the genealogy of *OsC1* might not be accurately reflected by the current distribution of alleles through lineage diversification.

## Materials and methods

### Plant Materials

Tainung 72 (TNG72), registered by Taiwan Agriculture Research Institute in 1987, is known as an aromatic elite cultivar. Taichung Sen 17 (TCS17), registered by Taichung District Agricultural Research and Extension Station in 1984, is one of two leading *indica* varieties in Taiwan. An F_2_ population of 624 individuals from a cross between these genotypes was planted in a paddy field at the Chiayi Agricultural Experiment Station (CAES), Taiwan, on January 21, 2008. The ratio of purple to green leaf sheath in the F_2_ population was 2.92: 1 (465:159), indicating single gene inheritance with green sheath being recessive. The 159 F_2_ individuals possessing GSH, which were predicted to be recessive homozygotes, were subjected to linkage analysis.

A panel of 50 accessions obtained from the National Plant Genetic Resources Center (NPGRC), Taiwan and The *Oryza* Map Alignment Project (OMAP) were used to reveal DNA sequence diversity of the gene conferring leaf sheath color. As detailed in Table 1, the panel included: (a) 43 accessions of Asian cultivated rice, comprised of 21 ssp. *japonica* accessions (8 cultivars, 13 landraces) and 22 ssp. *indica* accessions (9 cultivars, 13 landraces), and (b) 7 accessions of A-genome wild rice, including *O. rufipogon* (Taiwan type 1), *O. glaberrima* (96717), *O. barthii* (10412), *O. glumaepatula* (105668), *O. meridionalis* (105300), and *O. nivara* (2 accessions: 103813, 104680). Geographically, 26 accessions were from Taiwan (12 *indica*, 13 *japonica*, 1 *O. rufipogon*), 11 from China (7 *indica*, 3 *japonica*, 1 *O. nivara*), 5 from Japan (5 *japonica*), 2 from the Philippines (2 *indica*), 2 from India (1 *indica*, 1 *O. nivara*), and 1 each from Australia (*O. meridionalis*); Cameroon (*O. barthii*); Brazil (*O. glumaepatula*) and Senegal (*O. glaberrima*). Regarding leaf sheath color, 20 and 30 accessions exhibited purple and green, respectively.

### Measurement of Anthocyanin Contents

Total anthocyanin contents of leaf sheath and leaf blade harvested at four-leaf seedling and active tillering stages were quantified as described by Padmavati et al. (1997). Briefly, 0.5 g of fresh tissue was homogenized with extraction buffer and 1 % HCl/Methanol (V/V), for 24 h at 4 °C with occasional shaking. The supernatant was saved after centrifugation at 10,000 rpm for 15 min at 4 °C and absorbance at 530 nm measured using a spectrophotometer (Metertech SP8001, Taiwan). The relative contents of anthocyanin were estimated from A_530_ using an mM extinction coefficient of 31.6 (Padmavati et al. 1997).

### Genetic and Physical Mapping of the Gene Conferring Purple Sheath

For linkage analysis, 159 GSH F_2_ progeny, predicted to be recessive homozygotes, were genotyped with 121 polymorphic markers, including 67 SSR and 54 indel markers distributed over the 12 rice chromosomes. SSR primer sequences were retrieved from Gramene (http://www.gramene.org). The 54 indel markers were newly designed based on sequence divergence between Nipponbare (*japonica*) and 93–11 (*indica*) (Additional file 1: Table S1). DNA extraction and genotype assays of PCR-based markers was as described (Hsu et al. 2014). The linkage map was constructed with a LOD threshold of 3.5 by using MapMaker Exp 3.0 (Lander et al. 1987). The physical map of the target interval containing candidate genes was retrieved from the Rice Genome Annotation Project (RGAP, http://rice.plantbiology.msu.edu).

### Gene Expression of *OsC1*

Four accessions displaying various degrees of PSH were subjected to quantitative real-time PCR (qPCR) to investigate the relationship of *OsC1* gene expression with anthocyanin contents. These four accessions were *japonica* rice, Tainung 67 (TNG67, green), Tainung 72 (TNG72, light purple), Shang Chi Tsao Tao (SCTT, purple), and Kun Shan Wu Siang Keng (KSWSK, dark purple). Total RNA was extracted by using TRIZOL (Invitrogen, USA), and contaminating DNA was removed by using a TURBO DNA-free kit (Ambion, USA), according to manufacturer’s protocols. Primers of *OsC1* cDNA for qPCR were designed based on the gene sequence of GenBank accession number Y15219, and were forward primer: 5′-CAACgAgCTggTTTgAggCggT-3′ and reverse primer: 5′-TgAgAgACCACCTgTTgCCgAg-3′. Quantitation of gene expression and calculation of relative gene expression were as described (Hsu et al. 2014).

### Genetic Diversity Assessment

A panel of 50 accessions (above) possessing a range of leaf sheath colors were used to assess genetic diversity of *OsC1*. The *OsC1* DNA sequences of 1,311 bp were amplified by use of KOD plus *Taq* polymerase (TOYOBO, Japan) and Kapa Hifi DNA polymerase with three sets of primers, set 1 (F: 5′-ACATCgTACggggCTACAAg-3′, R: 5′-AgCgTTAgCCAgCTTCAAAT-3′), set 2 (F: 5′-ACTATCTCCggCCTAACATCAA-3′, R: 5′-TAgTAgTCgCAgTCgACgTC-3′), and set 3 (F: 5′-ATgTTgTCAggTggTCTCTC-3′, R: 5′-CACgTT CATgCAACCTTTTg-3′). The amplicons were sequenced using an ABI3730 DNA Analyzer following manufacturer’s protocol. The sequences were assembled and then subjected to multiple alignments using DNAMAN ver. 6.0 (Lynnon Biosoft) and consequently MAGA ver. 6.0 (Tamura et al. 2013).

Genetic divergence of the *OsC1* gene region was estimated with π (average nucleotide diversity per site, Nei and Li 1979) and θ_w_ (number of segregating sites, Watterson 1975) according to DnaSP version 5.10.1 (Librado and Rozas 2009). Two neutrality tests, 1) Tajima’s D and 2) Fu and Li’s D* & F*, were conducted to investigate whether there was any deviation from neutrality by using DnaSP version 5.10.1. Tajima’s D estimates the difference between the mean pairwise differences (π) and Watterson’s estimator (θ_w_) (Tajima 1989). Fu and Li’s D* & F* test reveals the discrepancy between the number of polymorphic sites in external and internal groups (Fu and Li 1993). A haplotype network, based on the probability of parsimony and calculated for pairwise differences until it exceeds 0.95, was constructed using TCS1.21 (Templeton et al. 1992).

## Additional file

Additional file 1: Table S1. Newly designed markers used in coarse mapping. (DOCX 17 kb)
