# Supplementary material for: Genetic and Evolutionary Analysis of Purple Leaf Sheath in Rice
Source: Rice (N Y). 2016 Feb 27;9:8. doi: 10.1186/s12284-016-0080-y (PMC4769704; doi:10.1186/s12284-016-0080-y)
Supplement: Additional file 1: Table S1. — Newly designed markers used in coarse mapping. (DOCX 17 kb) [file 12284_2016_80_MOESM1_ESM.docx]

**Additional file 1: Table S1: Newly designed markers used in coarse mapping**

| **ch** | **Primer** | **Forward primer sequence** | **Reverse primer sequence** |
| --- | --- | --- | --- |
| 1 | S13774 | TgATCAgCTTggACgTggTg | CAATgCCCgTAgTCTCTCAg |
| 1 | C62003 | CgggAggAAgATgACgATAg | TAgCTgAAAgTgCCACAATC |
| 1 | C52409 | TgTAACAgCAATATAggAgC | gTgCATTTgTTCAgTgATAC |
| 1 | E30745 | AAgAAgCAggAgAgTCgTgg | ATAATggATggTTCggTgCC |
| 3 | S00042 | CTAgggTCTTCTATggAAgC | CTgACCATgACATTgTAAgC |
| 3 | S02188 | CATTCTCCgggCAATTATTC | AATggCCTTCCTAAAACCTg |
| 3 | C53358 | ggAggAATgAATgAATgAAg | gCAgTATTCATACATAggAg |
| 3 | SLS178 | AgAAgAgTTACCTCCATCCT | AggCTTCAAgTTATTgCTAA |
| 4 | C61009 | ggCCAgCAAggTgTAgTAAg | ACAAACCCCAgCACCCTAAg |
| 4 | SLS189 | ATCAgAATATTCgggAAAAg | TTgTATACTCATTATgTAAATggA |
| 5 | E3528 | gggAAgAAgAgTAgAggCTg | TACACCCCAATATACCTTCg |
| 5 | E60663 | TCTTTgCCATggggggATAC | gCTgCTgCATAACAACATTC |
| 6 | CH0637 | CTgATTgCTCACTACTTCAT | ACCATggTCTAAAggTTCAg |
| 6 | CH0639 | CTTTAATTTTggACgTgCTT | gCTTTggACTgATTATgAgC |
| 6 | CH0611 | CgACgCTgTCAgAgACTgAg | gTCCAgCgTggTAgTCCAgT |
| 6 | CH0613 | CCACgAAgAACTgTAggACAA | ggTTTATCgTTgCTCATAAAAA |
| 7 | SLS164 | CTgCATATTTTCCCCTATTA | ggACAAggCACTAATACAgT |
| 7 | C53905 | gTAACggTAgCAgCAgCCAg | AgAAAACAAgCTgCCggATg |
| 8 | CH0866 | CTCCTTTCCCAATCTTACCT | gCgCATgCAgTATTATgTTA |
| 8 | SLS182 | ATCCTgACCTCTTgTTCTAC | TTAACATAgAAgACCATACgC |
| 8 | S20234 | CCATACACAACCACATTgCC | TgCTTgTATTAAACTgCCCg |
| 8 | SLS188 | ggCATCAgTAAgACACAATA | AATgAATCTgTCTAgATTgg |
| 8 | CH0862 | gAAgACgAgTgAggTCAgAA | TCCAATAAAACTgAggCTgT |
| 9 | S12569 | CACTgTATAgTACATCTTgg | TTgTAAAggATTggAgAAgg |
| 9 | SLS506 | ATCTCTCTAATCTTgCTggCT | TCCgTAACCCAAATAAACATA |
| 9 | SLS510 | TCAATTTgTgggTTAggTTTA | AACTgTgATTATCAACACgC |
| 9 | E21191 | CCCCTCgCTgAggCTTAATC | TCggCggCATAAAgCTgTAg |
| 10 | C51124 | CACTTCAgTgCTgggTgTgC | TCAAAgggCAAgTTAACgAC |
| 11 | CH1105 | TCgTTTCCTTCAAAACCTTA | TTCggTTgAACTgATAAATgT |
| 11 | S21074 | TgCTATAggTggTggTATgC | TTTCAAgCTgACgAACgATg |
| 12 | S13775 | TgATCAgCTTggACgTggTg | CAATgCCCgTAgTCTCTCAg |
| 12 | C42815 | CgggAggAAgATgACgATAg | TAgCTgAAAgTgCCACAATC |
| 12 | C33221 | TgTAACAgCAATATAggAgC | gTgCATTTgTTCAgTgATAC |
| 12 | E30746 | AAgAAgCAggAgAgTCgTgg | ATAATggATggTTCggTgCC |
